# TonB-Dependent Receptor Protein Displayed on Spores of *Bacillus subtilis* Stimulates Protective Immune Responses against *Acinetobacter baumannii*

**DOI:** 10.3390/vaccines11061106

**Published:** 2023-06-16

**Authors:** Nor-Aziyah MatRahim, Kathryn Marie Jones, Brian P. Keegan, Ulrich Strych, Bin Zhan, Hai-Yen Lee, Sazaly AbuBakar

**Affiliations:** 1 Tropical Infectious Diseases Research and Education Center (TIDREC), Universiti Malaya, Kuala Lumpur 50603, Malaysia; noraziyah@moh.gov.my (N.-A.M.); leehaiyen@um.edu.my (H.-Y.L.); 2 Texas Children’s Hospital Center for Vaccine Development, Baylor College of Medicine, Houston, TX 77030, USA; kathrynj@bcm.edu (K.M.J.); bkeegan@bcm.edu (B.P.K.); strych@bcm.edu (U.S.); bzhan@bcm.edu (B.Z.); 3 Virology Unit, Infectious Diseases Research Centre, Institute for Medical Research, National Institutes of Health, Shah Alam 40170, Malaysia

**Keywords:** infectious disease, antibiotic resistance, spores, oral vaccine, immunity

## Abstract

The emergence of antibiotic-resistant *Acinetobacter baumannii* strains with limited treatment options has become a significant global health concern. Efforts to develop vaccines against the bacteria have centred on several potential protein targets, including the TonB-dependent receptors (TBDRs). In the present study, TBDRs from *A. baumannii* were displayed on the surface of *Bacillus subtilis* spores. The immunogenicity of the recombinant spores was evaluated in orally vaccinated mice. None of the immunized mice demonstrated signs of illness and were observed to be healthy throughout the study. Sera and the intestinal secretions from the recombinant spores-treated mice demonstrated mucosal and humoral antibody responses to the vaccine antigen. In addition, bactericidal activities of the sera against *A. baumannii* clinical isolates were demonstrated. These observations suggest that the *B. subtilis* spore-displayed TBDRs should be further explored as much-needed potential oral vaccine candidates against *A. baumannii*.

## 1. Introduction

*Acinetobacter baumannii* is a nosocomial pathogen that causes a wide range of infections, including skin and soft-tissue infections associated with trauma, urinary tract infection, pneumonia, meningitis, and septicemia [1,2]. This bacterium, previously regarded as a low-grade pathogen, has acquired resistance against most of the commonly used antibiotics, marking the emergence of multidrug resistance (MDR) strains [2]. Currently, polymyxins, long considered agents of last resort, have become the chosen antimicrobial treatment for managing infections caused by this bacterium [3]. However, isolates that resist these “last resort” antimicrobials, known as extensive drug-resistant or XDR strains, have started to appear [4]. Owing to the pressing need for better treatment, the World Health Organization (WHO) has listed *A. baumannii* as a “Priority 1: Critical” agent for new and improved treatment options [5]. Since vaccination is recognized as a potentially effective alternative strategy to mitigate antimicrobial resistance (AMR), among the avenues explored is the development of *A. baumannii* vaccines [6].

Several studies have reported the potential of developing vaccines against *A. baumannii*, such as through formalin-inactivated whole cells (IWCs), containing outer membrane complexes (OMCs), outer membrane vesicles (OMVs), poly-N-acetyl-β-(1,6)-glucosamine (PNAG) [6], trimeric autotransporter protein (Ata) [7], K1 capsular polysaccharide (CPS) [8,9], or other outer membrane proteins such as OmpA, Omp22, and BamA [9,10,11]. The advancements in molecular technologies and whole-genome sequencing have contributed to the establishment of substantial whole-genome databases, allowing the rational design of vaccines using a process known as “reverse vaccinology” (RV). RV has already led to the discovery of previously unknown antigens and contributed significantly to our understanding of the biology of several pathogens leading to the development of universal vaccines for heterogeneous pathogens, as exemplified by the meningococcus B vaccine [12]. Several vaccine candidates for *A. baumannii* have been identified via RV, including FilF, BamA, OmpK, FK1B, OmpP1, and up to 8 TonB-dependent receptors (TBDRs) [13,14,15]. However, many of these candidates provide only partial protection against the infection.

Based on previous studies, the difficulty of achieving long-term protection using a single antigen has been acknowledged and antigenic combination approaches have been proposed [16]. The identification of antigens for a multivalent vaccine has been suggested to be based on (1) known physiological functions that are essential for the organism (known as the “siege” strategy) and/or (2) their involvement in the infection process (known as the “exhaustion” strategy) [17]. TBDRs are considered to function through the ‘siege’ strategy, triggering an immune response against key features of the bacterium, such as virulence, the acquisition of essential micronutrients, or being part of the outer membrane. Together with the fact that TBDRs are highly conserved among various *A. baumannii* strains has attracted interest in these antigens as vaccine candidates [17,18]. Among the TBDRs of *A. baumannii*, acinetobactin (BauA) and baumannoferrin (BfnH) have been identified as promising antigens for vaccines [19,20]. However, the likely low production yield of these outer membrane proteins hampered their further development [21]. To overcome this issue, here we utilized *Bacillus subtilis* spore surface display (BSSD) to produce the antigens. *B. subtilis* is classified as GRAS, or “*Generally Recognized As Safe*” by the US FDA, holding a safe record in human and animal use as a probiotic and food additive, and is permissible for use as a live bacterial vector [22,23]. It can form highly resistant dormant endospores under limited nutrient conditions and other environmental stresses [24]. Moreover, *B. subtilis* spores can simultaneously serve as an expression system and a delivery vehicle for the heterologous gene of interest, while also providing desired adjuvant properties [25].

Currently, most *A. baumannii* vaccine studies focus on the parenteral vaccination route, which generally elicits systemic antibody responses [26]. As the epithelial cells lining the mucosal surfaces are demonstrated to play a critical role in the host defence [9,10,11], vaccination via the mucosal route could potentially elicit a more effective immune response and confer better immune protection against the bacteria. The utilization of non-pathogenic bacteria classified as GRAS, e.g., *Lactococcus lactis* and *B. subtilis*, has been reported to elicit protective systemic and mucosal immune responses [27,28]. In the present study, we produced recombinant *B. subtilis* spores displaying *A. baumannii* TBDRs and evaluated their immunogenicity in orally fed mice.

## 2. Materials and Methods

### 2.1. Bacterial Origins, Plasmids, and Culture Conditions

A modified cry1Aa promoter derived from *Bacillus thuringiensis* was used to express and display the TBDRs on the surface of the *B. subtilis* spores [29]. The parental plasmid consisted of the cry1Aa promoter, the gene of interest (GOI), and the *cry1Ac* terminator, and was commercially synthesized by GenScript, USA. The naming of the gene of interest was based on the following: the organism, *A. baumannii*, denoted by *Ab*; the protein function, TonB receptor designated as TB; followed by the protein identifier. Thus, the target genes of interest were named AbTB6908 and AbTB1PR82, respectively. The DNA plasmids, pUC57 carrying the AbTB6908 and AbTB1PR82 genes, were commercially synthesized (GenScript, Piscataway, NJ, USA). The shuttle vector pHPS9 (ATCC 37816) was used as an expression vector for *B. subtilis*. All DNA manipulations were performed in *E. coli* One Shot^®^ Top10 cells (Invitrogen, Waltham, MA, USA). *B. subtilis* WB800N (MoBiTec, Göttingen, Germany) was used to express and display the recombinant GOI. As described elsewhere, *B. subtilis* sporulation was achieved by incubation in the GYS medium [30].

### 2.2. Construction of Recombinant B. subtilis Spores Displaying TBDR of A. baumannii

**Figure A1:**
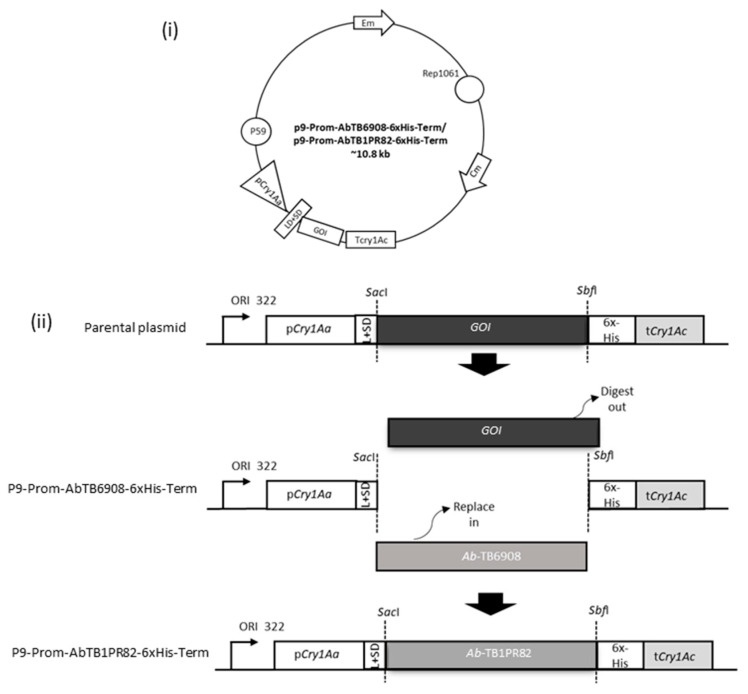
Schematic of the construct for TBDR display. Genetic map of the parental plasmid for the construction (**i**). Schematic of the construction of recombinant plasmids of pB-AbTB6908 and pB-AbTB1PR82 (**ii**).

Initially, a 6x-His sequence was inserted at the 3′-end of the AbTB6908 and AbTB1PR82 genes using a Q5^®^ Site-directed mutagenesis kit (NEB, Ipswich, MA, USA) with gene-specific primers (Table A1 in Appendix A). Later, the AbTB6908-6x His and AbTB1PR82-6x His genes were digested with the restriction enzymes SacI and SbfI, respectively. Concurrently, the MCS within the parental plasmid was digested with similar restriction enzymes and ligated with the AbTB6908-6x His and AbTB1PR82-6x His to generate pUC57-Prom-AbTB6908-6x His-Term and pUC57-Prom-AbTB6908-6x His-Term, respectively (Figure A1ii in Appendix B). The DNA fragments consisting of Prom-AbTB6908-6x His-Term and Prom-AbTB6908-6x His-Term were digested from pUC57 and ligated into a linearized shuttle vector, pHPS9 (named p9-Prom-AbTB6908-6x His-Term and p9-Prom-AbTB1PR82-6x His-Term, respectively). Finally, these two recombinant plasmids were transfected into *B. subtilis* WB800N according to the manufacturer’s protocol (MoBiTec, Göttingen, Germany). The recombinant *B. subtilis* strains were named Bs-AbTB6908 and Bs-Ab1PR82, respectively. *B. subtilis* transformed with the shuttle vector pHPS9 was included as a control and was named Bs-pHPS9.

### 2.3. Recombinant Spore Preparation and Evaluation of TBDR Protein on the Spore Surface

The recombinant *B. subtilis* sporulation was performed in the GYS medium at 37 °C for 24 h. Spore purification was performed as previously described [31], consisting of centrifuging cells for 10 min at 10,000× *g* at 4 °C and washing with 1M KCl and 0.5 M NaCl. Cells were washed 4 times with ice-cold distilled water, followed by overnight incubation at 4 °C. The spores were harvested by centrifugation. The spore coat was then removed using 50 mM dithiothreitol (DTT) and 1% sodium dodecyl sulfate (SDS) at 70 °C for 30 min. The spore coat was harvested by centrifugation at 8000× *g* for 10 min. The supernatant containing the spore coat proteins was subjected to SDS-PAGE, followed by the immunoblotting assay. The recombinant spores from Bs-AbTB6908, Bs-Ab1PR82 and Bs-pHPS9 were named sp6908, spPR82 and spHPS9, respectively.

The surface display of AbTB6908 and AbTB1PR82 were also analyzed using immunofluorescence microscopy. The purified spores were washed with AP buffer (100 mM Tris-HCl, pH 7.4, 100 mM NaCl, 0.25 mM MgCl_2_). The spores were then blocked in AP buffer containing 3% BSA (APB buffer) for 1 h. Next, the spores were incubated with APB buffer containing the primary antibody (anti 6x His-tag antibodies) raised in mice (dilution 1:100) for 2 h at 4 °C. Following incubation, the spores were washed 3X with cold AP buffer. Later, the cells were fixed with 4% paraformaldehyde for 30 min at room temperature (RT), washed, and incubated with anti-mouse IgG Alexa conjugate (Alexa Flour^®^488 goat anti-mouse IgG; Invitrogen, Waltham, MA, USA) in APB buffer at dilution of 1:1000 on ice for 2 h. The spores were washed 3× with cold AP buffer followed by fixation. For visualization under an immunofluorescence microscope, a 10 µL aliquot of the immunostained spore suspension (1:100 dilution) was spread onto a glass slide and air-dried. Next, a coverslip was mounted to the dried spores with 10 µL of VECTASHIELD^®^ antifade mounting media (Vector Laboratories, Newark, CA, USA). The fluorescence images were acquired using a DeltaVision Deconvolution Microscope (GE Healthcare, Chicago, IL, USA). The deconvolution setting was performed using a conservative method (based on ratio). The images from the bright field and the fluorescence were merged using ImageJ software version 1.54. The number of fluorescent spores was determined by dividing the number of fluorescent spores by the total number of spores observed in a microscopic field. At least ten randomly picked microscopic fields were used, and the average number of cells expressing the recombinant protein was calculated as previously described [32].

### 2.4. Animals and Immunization Schedule

**Figure 1:**
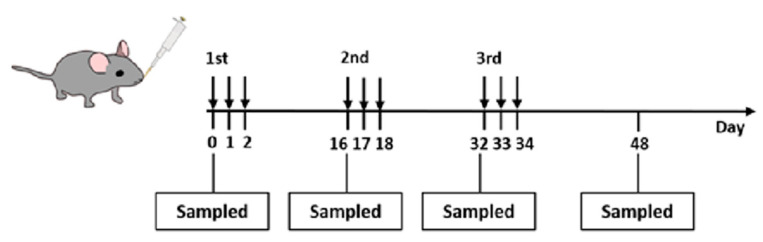
Schematic of the oral inoculation with recombinant *B. subtilis* spores and serum sampling. Mice were inoculated orally with 3 doses of recombinant *B. subtilis* spores displaying *A. baumannii* TBDR protein. The definition of 1 dose is 1 × 10^9^ CFU of recombinant spores daily for three consecutive days.

Adult female BALB/c mice between 8 to 10 weeks of age were purchased from Taconic Biosciences (Albany, NY, USA) and housed under specific pathogen-free conditions. The animal experiments were performed following the approved guidelines of Baylor College of Medicine, Institutional Animal Care and Use Committee, USA (Institutional Protocol Number: AN7511). The mice were randomly divided into three groups; *B. subtilis* spore expressed spHPS9 (control; n = 10), sp6908 (n = 10), and spPR82 (n = 10). Mice in the spHPS9 group served as the negative control. Mice were intragastrically administered with 1.0 × 10^9^ recombinant spores in 0.2 mL of PBS by intragastric lavage on days 0, 1, 2 (referred to as dose 1), 16, 17, 18 (referred to as dose 2), 32, 33, and 34 (referred to as dose 3). The mice were bled on days 0 (pre-bleed), 16 (week 2), 32 (week 4), and 48 (week 6) post-inoculation (PI), respectively. The mice were sacrificed on day 48. The intestines and livers of the mice were sterilely isolated, and the faeces were collected (Figure 1).

### 2.5. Sample Collection and Processing

Blood was obtained from the mice via tail vein bleeds. The harvested serum samples were stored at −20 °C until needed. The faecal pellet extracts (FPEs) were prepared as described earlier [33]. Faecal pellets (FPs) were collected from the housing cage of each mouse group. Briefly, the FPE was prepared from seven FPs by mixing with 600 µL of ice-cold PBS buffer with 0.1 mL of soybean trypsin inhibitor (STI) per ml, 1% (*w*/*v*) BSA, 25 mM EDTA, 1 mM PMSF, and 50% (*v*/*v*) glycerol. The mixture was allowed to emulsify at 4 °C for 4 h. After that, debris was removed by centrifugation at 15,500× *g* for 10 min at 4 °C. The supernatant was recovered into a microcentrifuge tube, pre-blocked with 1% BSA-PBS at RT for 2 h. The supernatant was kept at −20 °C until needed.

The intestines were harvested aseptically on day 48. The intestinal secretion from each mouse was extracted using the modified perfusion-extraction method (PERFEXT) described elsewhere [34]. Briefly, the intestine was washed twice with 3 mL of ice-cold PBS, followed by 2 times washing with 3 mL of ice-cold PBS containing 0.05 M EDTA, 0.1 mg/mL STI. The intestines were weighed before storage at −20 °C with PBS containing 2 mM PMSF, 0.1 mg STI/mL, and 0.05 M EDTA (1 mL/g tissue). The following day, the tissue was thawed, and saponin was added at 2% (*w*/*v*) final concentration to permeabilize the cell membranes. The samples were further incubated at 4 °C overnight. The mixture was centrifuged at 60,000× *g* for 20 min at 4 °C. The supernatant was recovered, and FBS was added to a final concentration of 3% before storage at −70 °C until needed.

### 2.6. Determination of TBDR-Specific Antibody by Indirect Spore ELISA

The assessment of antibody responses against the TBDR was performed using a modified spore ELISA method previously described elsewhere [35]. Briefly, the flat-bottom 96-well polystyrene plates (MaxiSorp; Nunc, Rochester, NY USA) were coated by passive absorption with 5 × 10^5^ CFU/well of recombinant spores expressing either AbTB6908 or AbTB1PR82 or BsS-pHPS9 in 1X coating solution (KPL, SeraCare, Milford, MA USA), overnight at 4 °C. The coated wells were fixed with 4% paraformaldehyde at 37 °C for 30 min, followed by 3 times washing with wash buffer; PBST (PBS, pH 7.4 with 0.1 % Tween 20).

After washing the plates, the wells were blocked with 1% BSA (SeraCare, Milford, MA USA) in PBST for 2 h at RT. Following the blocking step, the plates were washed 3 times with PBST. Before the test, the samples; serum (1:100), FPE (1:10), or mucosal secretion of mice (1:20), diluted in PBST with 0.1% BSA, were added into wells coated with BsS-pHPS9 and incubated for 2 h at RT (pre-adsorption). Following incubation, the samples were then added in duplicate into respective wells. The plates were incubated overnight at 4 °C. The wells were washed with 300 µL of PBST 3 times, and 100 µL of anti-mouse IgG/IgA HRP-conjugated secondary antibody was added at 1:4000 dilution for 1 h at 37 °C. The wells were washed 5 times with PBST, and the bound antibodies were detected by adding 100 µL of TMB microwell peroxidase substrate (KPL, SeraCare, Milford, MA, USA). The reaction was stopped after 1 h by adding substrate stop solution (KPL, SeraCare, Milford, MA, USA). The optical density (OD) values at 450 nm with 620 nm as reference wavelength was recorded. The data was analyzed and the cut-off value for a positive response for IgG and sIgA levels was calculated as described by Frey et al. [36].

### 2.7. Binding of Secretory IgA (sIgA) to A. baumannii Clinical Isolates

Three multidrug resistance clinical isolates, namely Ab16, Ab29, and Ab35, recovered from the skin, blood infection, and bronchoalveolar lavage (BAL), respectively, were used for the study (Table A2). The IgA binding assay was performed with the dot-blot and flow cytometry using freshly prepared bacteria culture. For the *A. baumannii* binding assay, the bacteria were inoculated into MH broth containing 2 μg/mL imipenem (IMP) and incubated overnight at 37 °C with continuous shaking at 180 rpm. The following day, the bacterial culture was inoculated into a fresh MH broth medium containing 2 µg/mL IMP (1:100 dilution). The inoculum was further incubated at 37 °C with continuous shaking at 220 rpm until it reached the late log phase (O.D600 = 0.8). Once the culture reached the designated absorbance reading, the bacterial culture was serially diluted to 1:100,000 in DPBS (equivalent to 150–250 live bacteria per 25 µL). *E. coli* (ATCC 25922 origin) was prepared by inoculating a single colony of the bacteria into lysogeny broth (LB; Oxoid, Thermo Fisher Scientific, Waltham, MA USA) and cultured at 37 °C with continuous shaking at 200 rpm overnight (Innova 44, New Brunswick, Edison, NJ, USA). Next, the bacterial culture was inoculated into a fresh LB medium (1:100) and further incubated at 37 °C with continuous shaking at 220 rpm until it reached the late log phase (O.D600 = 0.8). The culture was serially diluted, and the number of bacterial colonies was determined as described above for *A. baumannii*.

For dot-blot assay, approximately 1 × 10^6^ CFU of Ab16, Ab29, and Ab35 were dotted onto the nitrocellulose (NC) membrane and allowed to dry at RT. Three NC membranes were prepared for immunostaining with intestinal secretion from 3 mice groups. The dried NC membranes were blocked with 5% skimmed milk in 1X TBS at RT for 1 h. Following the blocking, the membranes were washed with 1X TBS containing 0.1% Tween-20 (TBS-T) 3 times, followed by the addition of the intestinal secretion from the group of mice orally fed with spHPS9, sp6908, and spPR82, respectively (1:100 dilution). The membranes were incubated overnight at 4 °C. The following day, the membranes were washed as described earlier, followed by the addition of anti-mouse IgA HRP-conjugated as a secondary antibody at 1:1000 dilution. The membrane was incubated with the secondary antibody at 37 °C for 1 h, followed by washing. The detection was performed using ECL™ Prime Western Blotting Detection Reagent (GE Healthcare, Chicago, IL, USA).

Flow cytometry analysis was performed using the three clinical isolates as described earlier. The bacteria cells (~1 × 10^6^ CFU) were initially washed in 500 μL of 1x PBS and fixed with 4% paraformaldehyde at RT for 30 min. The cells were then washed with 1x PBS, added with 1% BSA in PBS, and incubated at 37 °C for 30 min. Following that, the cells were washed twice before being added with the intestinal secretion from the group of mice orally fed with spHPS9, sp6908, and spPR82, respectively (1:100 dilution) and incubated overnight at 4 °C with constant rotation. The bacterial cells were washed, resuspended in 200 µL of anti-mouse IgA-fluorescein isothiocyanate (FITC)-labelled conjugate (1:1000 dilution; Southern Biotech, Birmingham, AL, USA), and incubated at 37 °C for 1 h. At the end of the incubation, the bacteria cells were washed and resuspended in 500 µL of 1x PBS. The cell suspension was transferred to a flow cytometry tube (5 mL) and assayed for antibody binding by flow cytometry using the BD FACSCanto™ II Flow Cytometer and BD FACSDiva™ Software (BD Biosciences, Franklin Lakes, NJ, USA). The bacterial events (10,000 events) were gated in the forward-scatter versus side-scatter plot to eliminate the “debris”. The antibody-bound-bacterium events were detected in the FITC channel and reported as percent positives or geometric mean fluorescence (GMF). The signal generated from cells stained with anti-mouse IgA FITC without prior incubation with the intestinal secretion served as the non-specific binding control. The data from the bacterial cell population incubated with the intestinal mucosal secretion of the mice group inoculated with sp6908 and spPR82 were normalized against the control mice orally fed with spHPS9 for analysis.

### 2.8. Complement-Mediated Bacteriolysis

Complement-mediated bacteriolysis was evaluated using *A. baumannii* clinical isolates, Ab16, Ab29, and Ab35. The *A. baumannii* clinical isolates were cultured as previously described (Method 2.7). Mice sera were heat-treated for 30 min at 56 °C to inactivate the complement. The heat-treated pooled mice sera (5%) were mixed with a 5% baby rabbit complement (Bio-Rad, Hercules, CA, USA). Later, the mice sera and baby rabbit complement mixture were added with the diluted Ab16, Ab29, and Ab35 cultures. The mixture was incubated at 37 °C for 1 h, followed by plating onto MH agar plates. The plates were incubated overnight at 37 °C, and the number of bacterial colonies formed was determined. The percentage of bacteriolysis was calculated as shown below:

$$\frac{\left(\begin{array}{c} No.of\;bacterial\;colonies\;after\;mixed\;with\;sera\;from\;control\;mice\;group-\\ No.of\;bacterial\;colonies\;after\;mixed\;with\;sera\;from\;vaccinated\;mice\;group \end{array}\right)}{\left(No.of\;bacterial\;colonies\;after\;mixed\;with\;sera\;from\;control\;mice\;group\right)}\times 100$$

### 2.9. Opsonophagocytic Killing Assay (OPKA)

The opsonophagocytic killing assay was performed using the mouse leukemic monocyte-macrophage cell line, RAW 264.7 (ATCC, Manassas, VA, USA), as previously described [37]. Briefly, the cells were seeded into a 96-well cell culture plate (Falcon, Fisher Scientific, USA) at a density of 2 × 10^4^ cells/well, followed by activation with 3-day exposure to 100 nM phorbol 12-myristate 13-acetate (PMA; Sigma-Aldrich, St. Louis, MO, USA). The *A. baumannii* clinical isolates Ab16, Ab29, and Ab35 were used as the target bacteria. *E. coli* was used as a control for potential cross-reactivity against Gram-negative bacteria. Both *A. baumannii* and *E. coli* bacterial cultures were prepared as described earlier. The test sera and intestinal mucosal secretions were diluted (1:10) before being added to the diluted bacterial culture’s aliquots. The mixture was incubated for 30 min at 37 °C and later used in the phagocytosis assay.

The opsonized bacteria were transferred to the macrophages prepared above for the phagocytosis assay. The cells were incubated for 1 h at 37 °C with 5% CO_2_ in a humidified incubator. The plates were gently agitated for 5 min at 15-min intervals. Following the incubation, the supernatant was recovered, plated onto MH agar plates, and incubated overnight at 37 °C. The number of bacterial colonies was determined, and the percentage of killing was calculated as previously described (Method 2.8). 

### 2.10. Statistical Analysis

Data were analyzed using GraphPad Prism Software (version 5) and expressed as the mean ± standard deviation (SD). The statistical analyses for sIgA antibodies were performed using paired t-tests. One-way analysis of variance (ANOVA) with Bonferroni post-test was used in the analyses of IgG antibodies. The bactericidal and OPKA activities were analyzed using two-way ANOVA with Bonferroni post-test.

## 3. Results

### 3.1. Construction of Recombinant Plasmids Carrying TBDR of A. baumannii

The display of TBDRs on spore surfaces was based on the expression of the target protein during the sporulation phase, as described elsewhere [30]. Here, two TBDR gene regions from *A. baumannii*, AbTB6908 (2132 bp) and AbTB1PR82 (2348 bp), were investigated as potential vaccine antigens. The *B. subtilis* expression vector was constructed using the plasmid carrying the *cry1Aa* promoter (529 bp), a Shine-Dalgarno sequence, and the leader sequence (SD + LD) from *B. thuringiensis cryIIIA* (558 bp, Figure A1i). The polyhistidine-tag sequence was placed in-frame, downstream of the TBDR gene region, to enable the detection of the recombinant proteins since no specific antibodies were available. Transcription of the gene from the promoter was terminated using the *cry1Ac* terminator fused downstream of the polyhistidine-tag sequence. The integrity of these constructs was verified by restriction enzyme digestion analysis and DNA sequencing.

### 3.2. Display of TBDR on the B. subtilis Spore Surface

**Figure 2:**
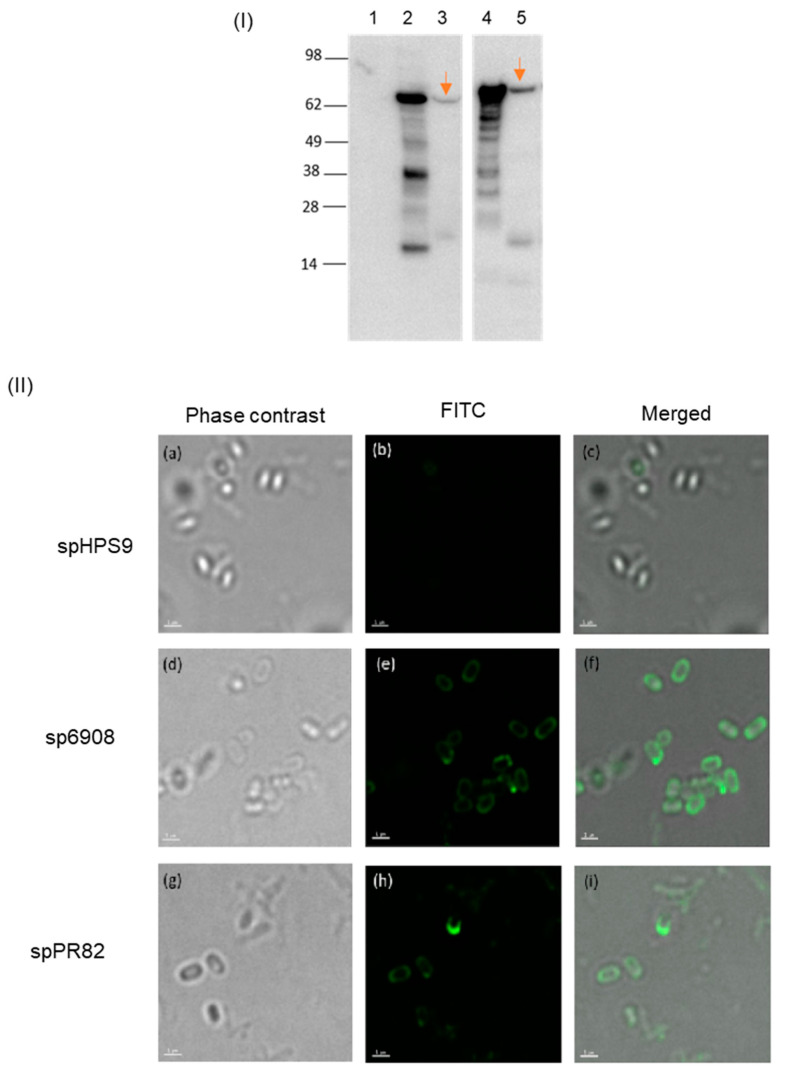
Expression of TBDRs on recombinant *B. subtilis* spores. The spore coat protein extract was separated on SDS-PAGE followed by immunoblot analysis (**I**). The arrows point to AbTB6908 (lane 3) and AbTB1PR82 (lane 5). The protein expressed in *E. coli* was included for comparison (lanes 2 and 4, respectively). Spore coat from BsS-pHPS9 was included as a negative control (lane 1). The expression of TBDRs on the spore surface was shown by immunofluorescence staining (**II**). Spores were fixed on slides and incubated with anti-His antibody and Alexa Flour^®^488 conjugated goat anti-mouse IgG. (Panels **a**,**d**,**g**) were observed under phase contrast, and (panels **b**,**e**,**h**) were visualized under fluorescence. (Panels **c**,**f**,**i**) are merged images of the fluorescence under phase contrast. All images were magnified 400-fold. Scale bars: 1 μm.

The expression of the TBDR proteins was initially evaluated using immunodetection. The extracted spore coat proteins were first separated on 4–12% SDS-PAGE gels, followed by immunoblotting. Protein bands of ~78 kDa and ~85.5 kDa representing AbTB6908 (Figure 2I, lane 3) and AbTB1PR82 (Figure 2I, lane 5), respectively, were detected by immunoblotting with the anti-His antibodies. These protein bands were comparable to the extracted inclusion bodies of *E. coli* cells expressing the same protein (AbTB6908: Figure 2I; lane 2; AbTB1PR82: Figure 2I; lane 4). No signal was detected in the extracts of *B. subtilis* transformed with an empty vector backbone, spHPS9 (Figure 2I, lane 1). The presence of TBDRs on the *B. subtilis* spore surface was further verified using immunofluorescence staining. The recombinant proteins AbTB6908 and AbTB1PR82 expressed on the surface of *B. subtilis* spore were observed with fluorescence microscopy using Alexa Flour 488 conjugated IgG secondary antibodies in combination with anti-6xHis antibodies (Figure 2II). No fluorescence signal was observed in the spores transformed with the empty vector BsS-pHPS9 control (Figure 2(IIb)). Microscopic examination reveals that the AbTB6908 and AbTB1PR82 proteins were present on the surfaces of 30% ± 2% and 24% ± 3% of the spores, respectively.

### 3.3. Animal Inoculation with the Recombinant Spores

In this study, for each inoculation, each mouse was inoculated with 1 × 10^9^ CFU of the recombinant spores. The dosage corresponds to the approved daily dose of GRAS by the US FDA for human consumption. No toxicity was observed in mice orally fed either with spHPS9 (control) or sp6908 and spPR82 spores. All mice were healthy throughout the study. The livers and intestines from immunized mice were examined macroscopically for any sign of toxicity, such as inflammation, discolouration, or cirrhosis. No signs of abnormality such as discolouration, enlargement or irregular/rough surface were observed in the examination of the livers. No sign of enlargement or swelling that reflects the inflammation was observed in any of the intestines. The mean weights of the livers were 1.138 ± 0.19 g, 1.034 ± 0.21 g, and 1.079 ± 0.18 g in those mice fed with spHPS9, sp6908, and spPR82, respectively (Table 1). The mean weights of the intestines of these mice were 2.105 ± 0.22 g, 1.942 ± 0.28 g, and 1.942 ± 0.28 g, respectively (Table 1). No significant differences were observed in the weights of the livers and intestines between the mouse groups fed with spHPS9 (control) or the group fed with recombinant spores, sp6908 and spPR82.

### 3.4. TBDR-Specific Antibodies in Inoculated Mice

**Figure 3:**
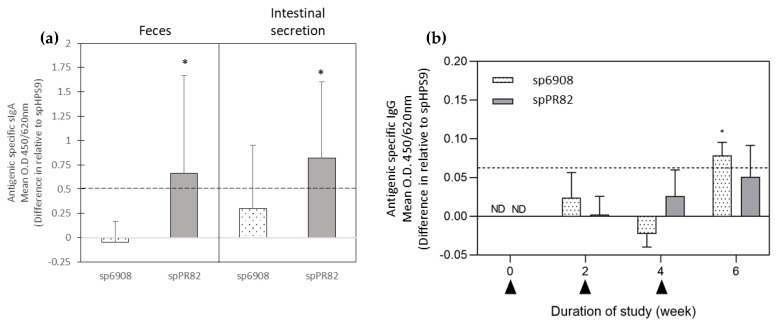
Antibody responses in mice orally fed with sp6908 and spPR82 were determined by indirect spore ELISA using recombinant spores, and the value was subtracted from the OD values of spHPS9 (control). Antigen-specific sIgA levels two weeks after the third oral feeding of mice with sp6908 and spPR82 were displayed as the difference in the mean OD value compared with the spHPS9 group (**a**). Kinetics of the IgG antibody responses in the pooled sera of mice orally fed with sp6908, spPR82, and spHPS9 were collected before the first feeding of each dose (**b**). The arrow represents the first, second, and third inoculation, respectively. The horizontal dotted line at 0.55 and 0.006 represent the cut-off for a significant positive response calculated as described by Frey et al. [36], for sIgA and IgG respectively. All data are expressed as the mean ± SD absorbance unit (* = *p* < 0.05).

**Figure 4:**
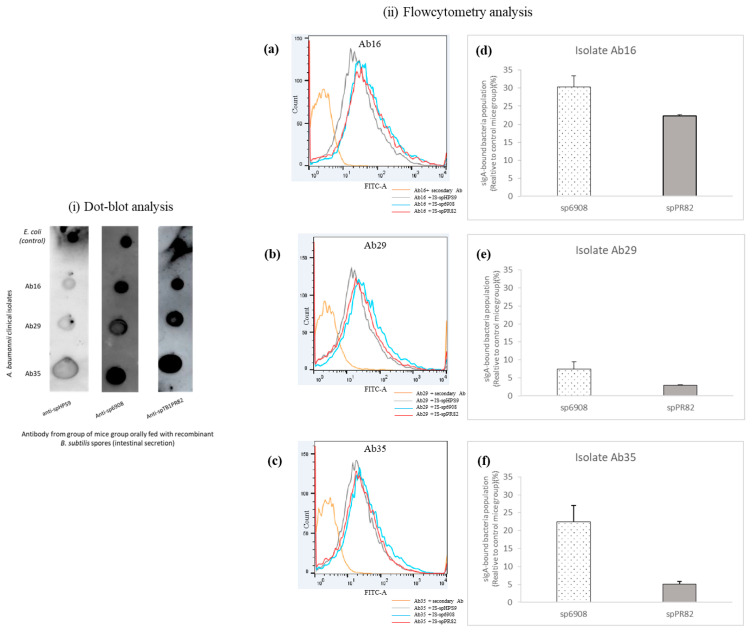
Binding of sIgA from intestinal secretions of orally vaccinated and non-vaccinated mice to MDR *A. baumannii* of different clinical origin. The interaction was evaluated by dot-blot (**i**) and flow cytometry (**ii**). The *A. baumannii* strains isolated from a skin lesion (Ab16), blood (Ab29), and BAL (Ab35) were blotted on the nitrocellulose membrane at a density of 1 × 10^6^ CFU and reacted with intestinal secretion from mice orally fed with recombinant *B. subtilis* spores. *E. coli* was used as an internal control as they are known to be commensal in mice guts. Using flow cytometry, the population of sIgA-bound *A. baumannii* clinical isolates was determined. The cell count versus fluorescence intensity histogram measured the binding of sIgA to Ab16 clinical isolate (**a**), Ab29 (**b**), and Ab35 (**c**). The percentage of sIgA-bound *A. baumannii* clinical isolates was generated after normalization against the signal obtained from the non-specific interaction between intestinal secretion of the control group, spHPS9 with the *A. baumannii* clinical isolates (**d**–**f**).

TBDR-specific sIgA antibodies in faeces and intestinal mucosa were analyzed using an in-house spore ELISA. The sIgA levels in the pooled faeces of mice inoculated with spPR82 were found to be statistically significantly higher than the spHPS9 mice group with a mean difference relative of 0.661 (t = 3.806, *p* = 0.0307; Figure 3a). No significant differences were observed in the sIgA levels of mice inoculated with sp6908 compared to the spHPS9 group (t = 0.1796, *p* = 0.8689; Figure 4). Additionally, the sIgA levels in the intestinal secretion of mice orally fed with sp6908 resulted in mean OD values of 1.109 ± 0.404 with a mean difference relative to spHPS9 of 0.289. The sIgA levels in the intestine of mice inoculated with spPR82 showed a statistically significant increase (t = 3.404, *p* = 0.0078) with a mean difference relative to spHPS9 of 0.8133 (Figure 3a). A cut-off value of 0.55 was obtained for the sIgA level assay. Based on the established cut-off value, the sIgA level in faeces and intestinal secretions of mice inoculated with spPR82 demonstrated a positive response of sIgA following inoculation with the recombinant spore.

The IgG levels in the mice sera inoculated with sp6908 were slightly increased in the absorbance reading two weeks after the first feeding compared to the control mice. The IgG levels in mice fed with sp6908 at week 4 showed no significant increase; the elevation in the antibody levels in this mice group was the highest with a statistically significant difference of 0.095, relative to the spHPS9 mice (control; Figure 3b). In the mice fed with spPR82, no significant difference in the IgG level compared to the control mice group was observed in the sera at week 2 (Figure 3b). At week 4 and 6, an increase in the IgG levels in the spPR82 mice was noted with a different mean OD relative to the spHPS9 group of 0.026 and 0.0514, respectively. However, the increase in IgG levels was not statistically significant (Figure 3b). A cut-off value of 0.06 was obtained for the IgG level assay. Referring to this value, sera of mice inoculated with sp6908 were observed to have a positive response of IgG at week 6 (2 weeks after the third dose).

### 3.5. Binding of sIgA from Intestinal Secretion to Clinical Isolates A. baumannii

Three different isolates of MDR *A. baumannii* recovered from patients’ skin (Ab16), blood (Ab29), and BAL (Ab35) were used in the study. The binding of sIgA from the intestinal secretion of mice fed with sp6908 and spPR82 to *A. baumannii* clinical isolates was demonstrated (Figure 4i). The sIgA binding levels of mice fed with sp6908 and spPR82 to the *A. baumannii* clinical isolates were comparable to the sIgA levels of the internal control, *E. coli*. A low correlation was observed between the sIgA levels from the intestinal secretion of the control mice group, BsS-pHPS9, with the *A. baumannii* clinical isolates (Figure 4i).

The binding of sIgA to *A. baumannii* clinical isolates was further demonstrated using flow cytometry. The antibody binding to the *A. baumannii* clinical isolates was quantified as a relative percentage compared to those obtained from the control mouse group inoculated with just the pHPS9 vector. The sIgA from the intestinal secretion of mice fed with sp6908 showed a higher percentage of binding to *A. baumannii* than those fed with spPR82 (Figure 4(iid–f)). The percentage of *A. baumannii* binding to sIgA varied between the clinical isolates. Clinical isolate Ab16 showed the highest binding, with 30.4% ± 3.0264% and 22.3% ± 0.26163% interaction with the sIgA from the mice group orally fed with sp6908 and spPR82, respectively (Figure 4(iid)). The lowest sIgA binding was observed for the clinical isolate Ab29 at 7.45% ± 2.008% and 2.995% ± 0.1626%, respectively (Figure 4(iie)). These results suggested differences in the binding of the antibodies from the intestinal secretion of the inoculated mice to the different *A. baumannii* clinical isolates.

### 3.6. Complement-Mediated Bacteriolysis Activity

Mice sera obtained on weeks 0, 2, 4, and 6 of the study were tested for the ability to promote complement-mediated bacteriolysis against the three different origins of MDR *A. baumannii*, as described above. The average number of the MDR *A. baumannii* colonies cultured on agar plates following treatment with the control mice (spHPS9) sera was 2.18 × 10^4^ ± 5.63 × 10^3^ CFU/mL, 1.68 × 10^4^ ± 7.30 × 10^3^ CFU/mL and 1.26 × 10^4^ ± 3.03 × 10^3^ CFU/mL for strains Ab16, Ab29 and Ab35, respectively.

**Figure 5:**
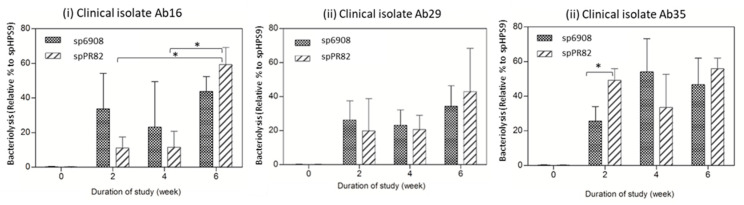
The complement-mediated bacteriolytic activity of heat-inactivated sera from mice orally fed with recombinant *B. subtilis* spores. The percentages of lytic activity were determined using sera from mice orally fed with sp6908 and spPR82 in the presence of baby rabbit complement against *A. baumannii* clinical isolates Ab16 (**i**), Ab29 (**ii**), and Ab35 (**iii**). The values were the means ± SD, tested in triplicate. The activities were analyzed using two-way ANOVA with Bonferroni post-test and *p* < 0.05 was considered significantly significant. (* = *p* < 0.05).

The percentage of bacteriolysis was presented as a relative percentage in comparison to the bacteriolysis effects observed in the sera of the control mice group. The bacteriolytic activity against clinical isolates Ab16 and Ab29 in mice sera fed with spPR82 demonstrated an increasing bacteriolysis percentage following the booster inoculation (Figure 5i–iii). A significant increase in the bacteriolytic activity was demonstrated in sera of the spPR82 mouse group against clinical isolate Ab16 (t = 54.93, *p* = 0.0077) (Figure 5i). However, a different pattern of bacteriolytic activity was observed against clinical isolate Ab35. A slight reduction in the killing activity was observed at week 4 (2 weeks after the booster); however, at week 6, a further increase in the bacteriolysis activity of 55.93% ± 6.08% was observed (Figure 5iii).

In the sera of mice fed with sp6908, a similar killing activity pattern to that observed in mice fed with spPR82 against clinical isolates Ab16 and Ab29 was noted. At week 2, the killing activity against clinical isolates Ab16 and Ab29 was 33.76% ± 20.53% and 26.29% ± 11.2%, respectively. A slight reduction in the bacteriolytic-killing activity was observed in week 4. Nevertheless, at week 6 (2 weeks after the second booster), an increase in the bacteriolytic activity in the sp6908 mice group was observed. The killing activity against Ab35 increased from 25.63% ± 8.42% (week 2) to 54% ± 19.23% (week 4). However, the killing activity at week 6 after the booster dose was only 46.63% ± 15.31% (Figure 5iii). Bacterial strain-dependent effects were observed in both mice groups, sp6908 and spPR82. The highest bacteriolytic activity was observed against clinical isolate Ab16 isolated from the skin, and the lowest killing activity was seen against the Ab29 isolated from a blood infection.

### 3.7. Opsonophagocytic Killing (OPK) Activity against Different Clinical Isolates

The opsonophagocytic killing (OPK) activity of sera and intestinal secretions from the inoculated mice were evaluated against the three MDR *A. baumannii* clinical isolates Ab16 (i), Ab29 (ii), and Ab35 (iii). RAW 264.7 cells were used in this assay.

**Figure 6:**
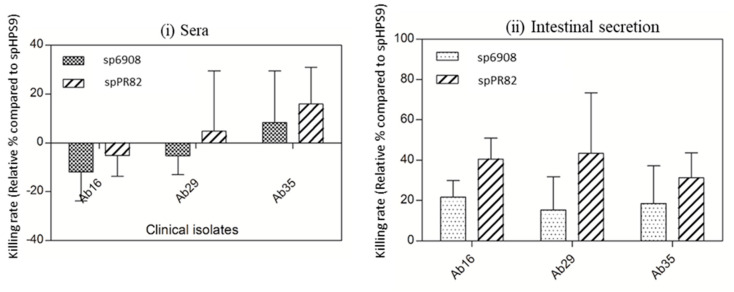
Opsonophagocytic killing activities in complement-inactivated sera and intestinal secretion of mice orally fed with recombinant *B. subtilis* spores against *A. baumannii* clinical isolates. Percentage of opsonophagocytic killing activity following opsonization with complement-inactivated sera (**i**) and intestinal secretion (**ii**) from mice orally fed with sp6908 and spPR82. The evaluation was performed against *A. baumannii* clinical isolates Ab16, Ab29, and Ab35, respectively. All the values were the mean ± S. D., tested in three independent assays. The activities were analyzed using two-way ANOVA with Bonferroni post-test and *p* < 0.05 was considered significantly significant.

Sera from mice fed with sp6908 showed no positive OPK effects against Ab16 and Ab29 isolates (Figure 6i). The sera showed 8.33% ± 21.12% killing activity against the clinical isolate Ab35 relative to the control group. The bactericidal activity in the complement-inactivated sera of the mice group fed with BsS-AbTB1PR82 demonstrated 4.8% ± 24.68% and 15.99% ± 15.05% OPK effects against Ab29 and Ab35, respectively. No positive OPK activity was observed in the sera of the mice group orally fed with spPR82 against the clinical isolate Ab16.

The OPK properties within the intestinal secretions showed that the intestinal secretions from the mouse group inoculated with spPR82 had killing activities against Ab16, Ab29, and Ab35 at 40.54% ± 10.38%, 43.4% ± 30.05%, and 31.3% ± 12.36%, respectively (Figure 6ii). The OPK activities in the intestinal secretion of mice inoculated with spPR82 were observed to have higher killing activities than those inoculated with sp6908 (*p* = 0.0331). It was also observed that the OPK activities in the intestinal secretion of inoculated mice demonstrated prominent killing activities compared to those observed in the complement-inactivated sera.

## 4. Discussion

The present study utilized the BSSD technique to produce the TBDRs of *A. baumannii* as an alternative expression method to overcome the issues of expressing the TBDR proteins in an *E. coli* expression system. Here, we have constructed the BSSD system without fusion to an anchor protein such as CotB, CotC, or CotG of *B. subtilis*; instead, with the presence of pCry1Aa, leader sequence, and the Shine-Dalgarno (SD) sequence from *B. thuringiensis*. We were able to display proteins with a molecular mass of >70 kDa with different protein properties on the surface of *B. subtilis* spores using the “copy-and-paste” approach. The produced recombinant spores were fed to mice without causing any sign of illness. The inoculation with recombinant spores sp6908 and spPR82 demonstrated elicitation of mucosal and systemic immune responses.

With *A. baumannii* becoming a serious global health threat, the development of new treatment and prevention measures against the MDR *A. baumannii* are urgently needed. Vaccines are recognized as potentially effective tools to mitigate antimicrobial resistance (AMR) [38]. Surface proteins or OMPs such as TBDRs are considered attractive vaccine targets as it is critical to bacterial virulence [39]. TBDRs play important roles in the transportation of necessary micronutrients (i.e., iron), pathogenicity, and immunogenicity of bacteria [18,40]. Among the TBDRs of *A. baumannii*, BauA is the most studied and demonstrated to be the ideal target for vaccine development. However, the commercial production of TBDR is challenging as they normally lead to lower expression yields. High levels of TBDR protein expression can be attained as inclusion bodies in the cytoplasm of *E. coli* [21], as observed in the expression of AbTB6908 and AbTB1PR82 in our study. Nevertheless, the removal of endotoxin and the refolding process resulted in tremendous protein losses. Our finding is in accord with other studies which demonstrated that most of the other OMPs did not fold efficiently, thus affecting their stability [41]. Using the BSSD technique, the heterologous proteins displayed on the spore surface did not require to go through a trans-membrane activity, thus avoiding incorrect folding and ensuring the structure and biological activity of the protein [42]. While it is not presently known if the recombinant proteins were displayed in their native configuration, earlier studies have demonstrated the successful display of other recombinant proteins in their native forms on the spore surfaces [30,43]. Results from the SDS-PAGE separation combined with the immunoblotting assay showed that the recombinant proteins AbTB6908 and AbTB1PR82 were successfully expressed with the demonstration of protein bands of similar sizes to the theoretical size of 78 kDa and 85.5 kDa, respectively. Immunofluorescence staining further confirmed the localization and expression of the proteins to the surface of the recombinant spores. A quantitative assay to determine the percentage of fluorescence spores over the non-fluorescence spores using flow cytometry was not performed due to the limitation of available flow cytometry to detect small particles such as spores. Nevertheless, the immunofluorescence microscopic analysis demonstrated that the expression of target proteins on the spores’ surface was at 30% and 24% for sp6908 and spPR82, respectively.

Previous studies on vaccine development for *A. baumannii* mainly focused on the parenteral route of administration. As the bacterium could infect many tissues with various routes of entry, including mucosal surfaces, oral delivery of vaccines could provide alternative stimulation of the protective immune response. The oral route is appealing as it avoids needles and potentially improves vaccine acceptance and compliance [44]. Since the gastrointestinal tract (GIT) is an important site for regulating the entrance of nutrients and responses toward foreign materials, it is believed that elicitation of prophylactic immunity at the site of infection entry could prevent infections [44,45]. Understanding the mucosal immune system and probiotics led to the development of a food-grade host/vector system (probiotic vector) as an antigen delivery vehicle for the oral vaccine [46]. Probiotics were proven to exert positive immunomodulation functions, such as enhancing secretory IgA (sIgA) and homeostasis of the immune system [47]. Amongst the studied probiotics utilized as host/vector systems include *Lactobacillus casei*, *Lactobacillus lactis*, the non-pathogenic strain of *E. coli*; *E. coli Nissle* 1917, and *B. subtilis* [27,48,49,50,51].

In the present study, all mice that intragastrically had received the recombinant *B. subtilis* spores showed no abnormalities or inflammation in their livers and intestines. Here, the IgG and sIgA levels were evaluated using in-house spore ELISA. The refolded TBDR proteins expressed in *E. coli* were initially evaluated as the coating antigen. However, the utilization of those proteins did not lead to success in the detection of antibodies. This could be due to the disruption in their conformational epitope. Since the antibodies elicited in mice were based on the conformational epitope on the BSSD, differences in the conformation of protein expressed in *E. coli* affected the recognition of antibodies towards the epitope [52]. Due to difficulties in obtaining refolded purified recombinant protein from the *E. coli* expression system, the recombinant spores were used as the coating antigen. Although the pre-adsorption step was performed, the background signal was considerably high, which may affect the sensitivity of the assay in assessing the antibodies levels. Nevertheless, the differences in the specific sIgA and IgG levels could be assessed and demonstrated. The specific sIgA levels in the faeces and intestinal secretion increased, especially in those administered with the spPR82 recombinant spores. The increment in the specific IgG levels in sera of mice fed with *B. subtilis* displaying TBDR suggested that the recombinant spores successfully elicited both mucosal and systemic immune responses. The function of the elicited antigen-specific antibodies in the sera and intestinal secretion was also assessed.

The interaction between the sIgA antibodies was found in the intestinal secretion of mice fed with the recombinant *B. subtilis* spores against three *A. baumannii* clinical strains isolated from a skin lesion (Ab16), blood (Ab29) and BAL (Ab35) was evaluated. Specific binding between the sIgA and *A. baumannii* clinical isolates was observed. Since low positive signals were obtained in the interaction between the *A. baumannii* clinical isolates and the sIgA from mice fed with spHPS9 control, it was deduced that the observed signals in the dot-blot assays were explicitly from the *A. baumannii* sIgA antibodies interaction. For this study, *E. coli* was used as an internal control in the dot-blot analysis, as the bacterium is a known mouse gut commensal [53]. Further evaluation of the sIgA binding to *A. baumannii* was performed using flow cytometry. It was observed that the sIgA binding was *A. baumannii* strain-dependent, with the lowest interaction noted against the clinical isolate Ab29. The isolate was initially recovered from the blood of a patient. It was previously reported that clinical isolates recovered from blood encoded 50 genes essential for complement resistance, with the most prominent function related to maintaining the outer membrane (OM) lipid asymmetry [54]. Thus, the low percentage of interaction between the sIgA and clinical isolate Ab29 could be attributed to the OM lipid asymmetry that disrupts the antibody binding interaction. On the other hand, for flow cytometry analysis, the gating area was set based on the unstained cells, where most bacterial cells were distributed as single cells. The low detection of binding could also be simply due to the sIgA-mediated bacterial clumping since it was previously reported that the sIgA could mediate bacterial clump formation [55]. We could have overlooked this possibility, and the percentage of sIgA-bound bacteria could have been underreported.

The present study also evaluated the effector function of antigen-specific antibodies against *A. baumannii* to promote complement-mediated bacteriolysis and immune phagocytosis (opsonization). Results from the study showed that the antigen-specific IgG in the serum of mice fed with the recombinant *B. subtilis* spores promoted bacteriolysis via complement activation with killing activity up to 60% after three doses of oral feeding. The killing activity, however, was the lowest against the strain isolated from blood, Ab29. This observation was consistent with the earlier result from the antibody binding study between sIgA and the Ab29 isolate, highlighting the potential disruption of the antibody binding towards the proposed asymmetrical outer membrane (OM) [54]. From the complement-mediated bacteriolysis analysis, it was noted that although the IgG level in the mice group fed with spPR82 was lower than in the mice group fed with sp6908, higher bacteriolytic activity against *A. baumannii* clinical isolates was observed in the former group. Our finding suggests that despite the lower total IgG in the sera of mice fed with spPR82, the secreted IgG subtypes showed a higher capacity to induce complement-mediated bacteriolysis [56].

The opsonophagocytosis (OPK) properties of the recombinant spore-treated mice sera and the intestinal secretion were evaluated using murine macrophage-like cells, RAW264.7. Macrophages are important early defenders in promoting neutrophil recruitment and phagocytosis [57,58]. From this study, it was observed that the IgG antibodies in sera of mice fed with recombinant *B. subtilis* spores displaying the TBDR protein (particularly spPR82 mice group) demonstrated a higher killing rate (~10–20%) in comparison to the control group. These findings suggested that the serum IgG possessed opsonic properties to induce phagocytic killing. The OPK activity, however, could be higher in the presence of complement, as observed in another study on the OMV vaccine against *A. baumannii*, which demonstrated the role of complement-dependent OPK effects mediated by the antibodies. Evaluation of the OPK properties of antibodies within the intestinal secretion showed that the mice fed with the recombinant *B. subtilis* spores showed up to 40% killing activities. The killing activity could be contributed by the oxidative burst triggered by sIgA-opsonization, as reported in a few other studies on sIgA activities [59,60]. However, no measurement of the superoxide released by the macrophages was performed in this study.

The presence of specific TBDR antibodies demonstrated in the present study suggested that the recombinant *B. subtilis* spores displaying the TBDR protein could resist intestinal degradation and elicit mucosal and systemic antibody responses. It also suggests that the *A. baumannii*, AbTB1PR82 (2348 bp) is more immunogenic compared to AbTB6908 (2131 bp). While the AbTB1PR82 protein used in the study showed more promising results, there are about 21 other TBDRs that have been identified in *A. baumannii* [61] that are yet to be explored. A combination of the different TBDRs with other antigens that play a role during bacterial infection such as LptD and OmpA [17] using a multivalent approach could be explored in the future for enhanced elicitation of the protective immune responses.

The study presented here, however, has a few limitations that could be addressed in a future study. One major limitation is that the percentage of spores displaying the recombinant proteins was only around 24–30%. And since only 1 × 10^9^ CFU per feeding as it corresponds to the approved daily dose by the US FDA for human consumption was used, the number of actual recombinant spores fed to each mouse was much lower, at about 2.4–3 × 10^8^ CFU. Increasing the fraction of functionalized spores could perhaps significantly improve the immune responses and the effector functions of the antibodies. The study also did not take into consideration cellular immunity, including the cytokine profile. This was mainly due to the lack of available resources and reagents, especially the inability to obtain purified soluble and well-refolded recombinant TBDRs. Another limitation of this study is the lack of an animal challenge model for *A. baumannii* infection as the clinical isolates do not replicate in immunocompetent mice [26,62]. Although some hypervirulent *A. baumannii* strains, i.e., HUMC-1, AB5057, LAC-4, and VA-AB41, were reported to cause lung infection and bacteremia in mice, access to these bacterial strains was not available for this study. In the future, further immunogenicity evaluations could perhaps be performed in rats, as they have demonstrated to be a better animal model for *A. baumannii* than mice, as rats have many characteristics that mimic human infection [26].

In summary, in this study, two *A. baumannii* TBDRs, AbTB6908, and AbTB1PR82 were successfully expressed as recombinant proteins on the surface of *B. subtilis* spores. Mice fed with these recombinant spores produced antigen-specific immune responses in the intestinal mucosa as well as in sera. The sIgA of the mouse intestinal mucosa bound to *A. baumannii* clinical isolates, induced complement-mediated bacteriolysis, and mediated antibody-dependent cellular cytotoxicity. These results suggest that *B. subtilis* spores displaying *A. baumannii* TBDRs demonstrated promising oral vaccine properties against *A. baumannii* infection. Further evaluation of the safety profiles, ability to promote even more potent immune responses, its possible interaction with the normal commensals, and demonstration of protective immunity in a suitable animal challenge model, however, are still needed.

## 5. Patents

The reported work has been submitted to the Intellectual Property Corporation of Malaysia (MyIPO) for patent filing.

## Figures and Tables

**Table 1:** Weight of liver and intestine of mice inoculated with recombinant *B. subtilis* spores.

Groups	Liver (g)	Intestine (g)
Control (spPS9)	1.138 ± 0.193	2.105 ± 0.220
sp6908	1.034 ± 0.210	1.942 ± 0.228
spPR82	1.079 ± 0.175	1.942 ± 0.277

Data are expressed as mean ± SD for each group.

## Data Availability

Data presented in this study are available on request from the corresponding author.

## Appendix A

**Table A1:** List of primers used.

NAME	PRIMER SEQUENCE (5’-3’)
For confirmation of the insert in pHPS9 vector (sequencing)	
pHPS9_1000F	TTAAAAACATATATTTGC
pHPS9_3544R	CTAGTTGGTTTGTAGACG
407-R	CTAAATATCAATTTTTGACC
4783-F	CATGAGATCGAGAAC
For the generation of constructs with 6x-His sequence	
B.sub_all-F	CGAGCTC GGGGAGGAGAATCATG SacI
Ab6908BsubR	GCCTGCAGGTTAGTGATGGTGATGGTGATGAAAGCGATATCTAAGCG SbfI 6x His
Ab1PR82BsubR	GCCTGCAGGTTAGTGATGGTGATGGTGATGATAATTAAATGTATAGC SbfI 6x His
For insertion of a 6x-His sequence using site-directed mutagenesis	
Q56908_F	CACCACCACTAACCTGCAGGCATTCCA
Q56908_R	ATGATGATGAAAGCGATATCTAAGCGC
Q51PR82_F	CACCACCACTAACCTGCAGGCATTCCA
Q51PR82_R	ATGATGATGATAATTAAATGTATAGCTAAGGCC

## Appendix C

**Table A2:** Antibiotic resistance profile of MDR *A. baumannii* strains used in this study.

Isolates	Source	Antimicrobial Susceptibility Profile																		
		AP	SXT	SAM	AMC	CN	NET	CXM	CAZ	CRO	CTX	PIP	CIP	IPM	MEM	FEP	SCF	TZP	AN	CT
Ab16	Skin Blister	R	S	R	R	R	R	R	R	R	R	R	R	R	R	R	R	R	S	S
Ab29	Blood	R	R	R	R	R	R	R	R	R	R	R	R	R	R	R	R	R	R	S
Ab35	Bronchoalveolar Lavage	R	R	R	R	IN	R	R	R	R	R	R	R	R	R	R	R	R	R	S
